# Pembrolizumab‐Induced Anti‐Yo‐Antibody‐Positive Cerebellitis: A Case Report

**DOI:** 10.1002/rcr2.70160

**Published:** 2025-03-24

**Authors:** Takafumi Yamano, Toshihide Yokoyama, Tadashi Ishida

**Affiliations:** ^1^ Department of Respiratory Medicine Kurashiki Central Hospital Okayama Japan

**Keywords:** anti‐Yo‐antibody, cerebellitis, immune checkpoint inhibitor, immune‐related adverse event, pembrolizumab

## Abstract

We report a rare case of pembrolizumab‐induced anti‐Yo‐antibody‐positive cerebellitis in a patient with stage IV lung adenocarcinoma. Following two cycles of combination therapy, the patient developed nystagmus and ataxia. Despite tumour regression and normal magnetic resonance imaging (MRI) findings, cerebrospinal fluid analysis revealed lymphocyte‐dominant hypercellularity, increased protein, and oligoclonal bands. Immune checkpoint inhibitor (ICI)‐related cerebellitis was diagnosed after excluding infectious causes, metastases, and cancerous meningitis coupled with a positive anti‐Yo antibody. Methylprednisolone pulse was ineffective, but high‐dose intravenous immunoglobulin therapy resolved symptoms. To the best of our knowledge, this is the first case report of ICI‐related cerebellitis with anti‐Yo‐antibody in lung adenocarcinoma. This case highlights the importance of paraneoplastic antibody testing in diagnosing ICI‐related encephalitis, especially in cases of normal MRI findings.

## Graphical abstract

We report a rare case of pembrolizumab‐induced anti‐Yo‐antibody‐positive cerebellitis in a patient with stage IV lung adenocarcinoma. To the best of our knowledge, this is the first case report of ICI‐related cerebellitis with anti‐Yo‐antibody in lung adenocarcinoma. This case highlights the importance of paraneoplastic antibody testing in diagnosing ICI‐related encephalitis, especially in cases of normal MRI findings.

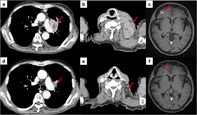

## 1 Introduction

Pembrolizumab, an anti‐PD‐1 monoclonal antibody, can cause immune‐related adverse events (irAEs), including cerebellitis. Immune checkpoint inhibitor (ICI)‐related encephalitis often involves paraneoplastic antibodies, such as anti‐Yo antibody, which is associated with cerebellar degeneration in ovarian, breast, and small cell lung cancers, but rarely in lung adenocarcinoma. We report the first case of pembrolizumab‐induced cerebellitis with anti‐Yo antibody in a patient with stage IV lung adenocarcinoma.

## 2 Case Report

**FIGURE 1:**
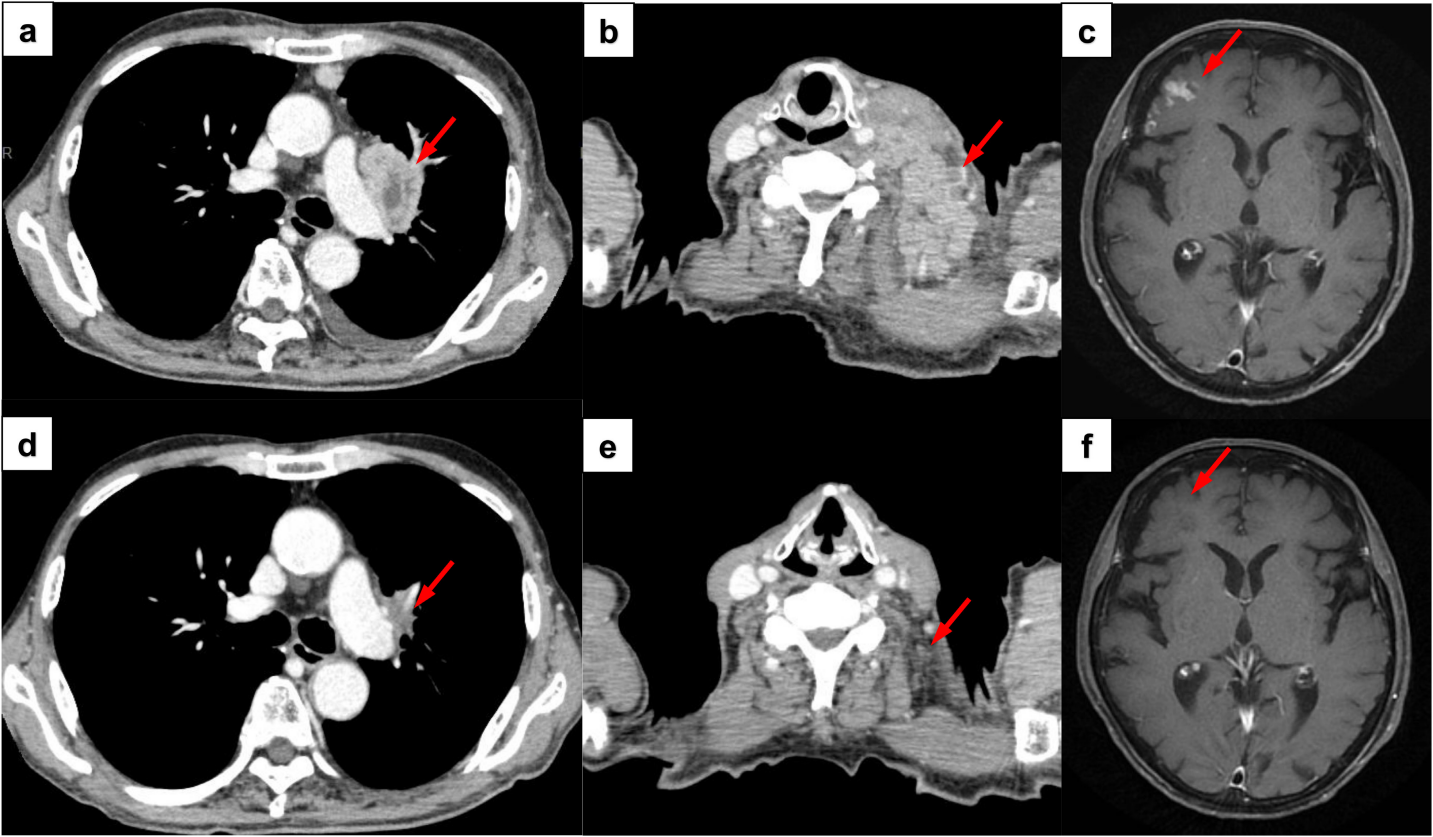
Imaging findings of the present case before and after two cycles of chemotherapy. Contrast‐enhanced computed tomography (CECT) scans before chemotherapy showed (a) lung cancer in the left upper lobe and (b) metastases to the left supraclavicular lymph nodes. (c) Contrast‐enhanced magnetic resonance imaging (MRI) showed brain metastases in the right frontal lobe. Images (d–f) represent the corresponding findings after two cycles of chemotherapy.

A 75‐year‐old man with an Eastern Cooperative Oncology Group Performance Status score of 1 presented to our hospital with a chief complaint of cough. He had a 53‐pack‐a‐year history of smoking. Computed Tomography (CT) showed a 42.1‐mm mass in the left upper lobe, left supraclavicular lymph node metastasis, and right frontal lobe brain metastasis, leading to a diagnosis of lung adenocarcinoma cT2bN3M1b Stage IVA based on the UICC TNM classification (8th edition) (Figure 1). Carboplatin, pemetrexed, and pembrolizumab were administered, but on day 13, after two cycles of administration, the patient presented to the emergency department with nausea and dizziness.

On presentation, the patient's vital signs were as follows: GCS E4V5M6; temperature, 36.7°C; BP, 169/92 mmHg; HR, 93/min; RR, 16/min, and SpO_2_ 97% in room air. Physical examination revealed diplopia and bilateral vertical nystagmus. Pupillary examination revealed equal pupils (3 mm bilaterally) with normal contralateral light reflex. Extraocular movements, visual fields, and other cranial nerve functions were intact. Meningeal signs were absent. However, the patient exhibited positive Romberg's sign, impaired tandem gait, and a wide‐based gait. Upper and lower extremity strength was bilaterally preserved (Manual Muscle Testing score: 5/5). Barre's sign, Mingazzini's sign, finger‐nose‐finger test, and knee–heel test were negative. Blood test findings showed no notable abnormalities (Table 1).

**TABLE 1:** Laboratory findings.

	Value	Reference range		Value	Reference range		Value	Reference range
Blood count			Glucose (mg/dL)	117	70–109	Anti‐titin antibody	(−)	(−)
WBC (/μL)	3000	3300–8600	HbA1c (%)	5.9	4.6–6.2	Anti‐zic4 antibody	(−)	(−)
Neutrophil (%)	63.5	38.0–77.0				Anti‐GAD65 antibody	(−)	(−)
Lymphocyte (%)	27.6	15.0–53.0	Immunology			Anti‐Tr antibody	(−)	(−)
RBC (×10^4^/μL)	411	4.35–5.55	IgG (mg/dL)	1644	861–1747			
Hb (g/dL)	9.2	13.7–16.8	RF (IU/L)	7.6	< 15	Influenza antigen	(−)	(−)
Plt (×10^4^/μL)	37.3	16.0–36.0	ANA (titre)	< 40	< 40	SARS‐CoV‐2 PCR	(−)	(−)
Biochemistry			PR3‐ANCA (IU/mL)	1.2	< 2.0	Entero/Rhino virus PCR	(−)	(−)
CRP (mg/dL)	0.12	0.00–0.14	MPO‐ANCA (IU/mL)	< 1.0	0.0–3.4			
TP (g/dL)	6.6	6.6–8.1	Anti‐TPO antibody (IU/mL)	11	< 16	Cerebrospinal fluid		
Alb (g/dL)	3.2	4.1–5.1	Anti‐Tg antibody (IU/mL)	14	< 28	Initial pressure (mmH_2_O)	180	75–150
T‐Bil (mg/dL)	0.3	0.4–1.5	CMV IgM (S/CO)	0.08	< 0.85	Cell count (/μL)	26	0–5
AST (IU/L)	19	13–30	CMV IgG (AU/mL)	218	< 6.0	Lymphocyte (%)	96	
ALT (IU/L)	21	10–42	HSV IgM (EIA)	< 0.8	< 0.8	Neutrophil (%)	4	
γ‐GTP (IU/L)	51	13–64	HSV IgG (EIA)	63.2	< 2.0	Atypical cells	(−)	(−)
LDH (IU/L)	155	124–222	VZV IgM (EIA)	< 0.8	< 0.8	Protein (mg/dL)	70	10–40
CK (IU/L)	26	59–248	VZV IgG (EIA)	22.3	< 2.0	Glucose (mg/dL)	71	50–75
Creatinine (mg/dL)	0.73	0.65–1.07	Anti‐Yo antibody	(+)	(−)	IgG‐index	2.7	< 0.8
Sodium (mEq/L)	134	138–145	Anti‐AMPH antiobody	(−)	(−)	Oligoclonal band	(+)	(−)
Potassium (mEq/L)	4.4	3.6–4.8	Anti‐CV2 antibody	(−)	(−)			
Calcium (mg/dL)	8.5	8.8–10.1	Anti‐PNMA2 antibody	(−)	(−)			
TSH (μIU/mL)	1.21	0.38–5.38	Anti‐Ri antibody	(−)	(−)			
FT4 (ng/dL)	1.24	0.70–1.48	Anti‐Hu antibody	(−)	(−)			
Vitaimin B1 (ng/mL)	26	26–58	Anti‐recoverin antibody	(−)	(−)			
Vitamin B12 (pg/mL)	612	197–771	Anti‐SOX1 antibody	(−)	(−)			

Abbreviations: γ‐GTP, gamma‐glutamyl transpeptidase; Alb, albumin; ALP, alkaline phosphatase; ALT, alanine aminotransferase; ANA, anti‐nuclear antibody; AST, aspartate aminotransferase; CK, creatine kinase; CMV, cytomegalovirus; CRP, C‐reactive protein; FT4, free T4; Hb, haemoglobin; HbA1c, haemoglobin A1c; HSV, herpes simplex virus; IgG, immunoglobulin; LDH, lactate dehydrogenase; MPO‐ANCA, myeloperoxidase‐anti‐neutrophil cytoplasmic antibodies; Plt, platelet count; PR3‐ANCA, proteinase‐3‐anti‐neutrophil cytoplasmic antibodies; RBC, red blood cell count; RF, rheumatoid factor; T‐Bil, total bilirubin; Tg, thyroglobulin; TP, total protein; TPO, thyroid peroxidase; TSH, thyroid stimulating hormone; VZV, varicella zoster virus; WBC, white blood cell count.

CT showed lung cancer regression. Magnetic resonance imaging (MRI) of the brain showed loss of contrast enhancement in the right frontal lobe and no evidence of acute cerebral infarction or encephalitis (Figure 1). Cerebrospinal fluid (CSF) analysis indicated lymphocyte‐dominant pleocytosis, elevated protein levels, the presence of oligoclonal bands, and an increased IgG index. However, Gram stain, Ziehl–Neelsen stain, and cytology were negative. As the possibility of viral infection could not be ruled out, the patient was treated with metoclopramide and betahistine mesylate for 1 week, but it was ineffective. MRI was repeated on day 8, but no indications of encephalitis or cerebral infarction were detected. Throat swab analysis yielded negative results for influenza antigen, SARS‐CoV‐2 PCR, and enterovirus/rhinovirus PCR. Serological testing indicated past infections with herpes simplex virus and herpes zoster virus, while antinuclear antibodies, anti‐ds‐DNA antibodies, anti‐SS‐A antibodies, and ANCA were negative. The paraneoplastic neurological syndrome antibody panel provided by BML Inc. was positive for the anti‐Yo antibody. ICI‐related cerebellitis was suspected, and steroid pulse therapy was initiated on day 9, followed by 50 mg of prednisolone (1 mg/kg). However, as the symptoms did not improve, high‐dose intravenous immunoglobulin (IVIG) was administered on day 14. Nystagmus, dizziness, and light‐headedness were reduced on day 29 and resolved completely on day 33. Prednisolone was tapered and discontinued on day 190, and the patient showed no recurrence. Subsequent anti‐Yo antibody testing was negative.

## 3 Discussion

ICI‐related encephalitis occurs in 0.2% of patients using ICI, with time to onset ranging from 18 to 297 days, with a median of 51.5 days [1]. In ICI‐related encephalitis, MRI findings are usually normal and CSF analysis typically reveals lymphocytic pleocytosis and elevated protein levels [1]. The findings in the present case were consistent with these features.

Differential diagnosis included acute infectious cerebellitis, post‐infectious cerebellitis, cerebellar metastases, and carcinomatous meningitis. Acute infectious cerebellitis, caused by Epstein–Barr virus, Varicella–Zoster virus, influenza, enterovirus, dengue virus, and JC virus [2], was ruled out based on negative serological and PCR results. The patient's history and MRI findings did not support post‐infectious cerebellitis, cerebellar metastases, or carcinomatous meningitis. Anti‐Yo‐antibody, or anti‐Purkinje cell cytoplasmic antibody type‐1 (PCA‐1), targets Purkinje cells and granule cells in the dento‐cerebellar cortex and is associated with paraneoplastic cerebellar degeneration. In patients with paraneoplastic cerebellar degeneration, this antibody is common in ovarian, breast, and small cell carcinomas, but rare in non‐small cell lung cancer [3]. Furthermore, ICI‐related cerebellitis with anti‐Yo‐antibody has not been reported in lung adenocarcinoma. ICI‐related neurological irAEs resemble paraneoplastic neurological syndromes, and paraneoplastic autoantibodies including anti‐Yo‐antibody are frequent [4]. Here, ICI‐related cerebellitis was diagnosed based on symptom development after ICI administration despite tumour regression, the presence of cerebellar ataxia, and the positive anti‐Yo antibody. Subsequent normalisation of anti‐Yo‐antibody levels after prednisolone treatment further supports its role in the pathogenesis.

ICI‐related encephalitis is managed by discontinuing ICI and methylprednisolone (1–2 mg/kg). If symptoms are severe or oligoclonal bands are present, methylprednisolone pulse (1 g for 3–5 days) and IVIG (400 mg/kg for 5 days) are required. For cases with positive paraneoplastic autoantibodies and poor treatment response, rituximab or plasma exchange may be considered as additional therapeutic options [5]. Here, IVIG was added after ineffective methylprednisolone therapy, leading to improvement.

This report has a limitation that the possibility of a false‐positive anti‐Yo antibody result could not be ruled out. However, as the patient presented with cerebellar ataxia, which is consistent with the clinical manifestations of anti‐Yo antibodies, we consider it unlikely to be a false‐positive result.

ICI‐related encephalitis may lack MRI abnormalities, making paraneoplastic autoantibody detection valuable. Therefore, screening for these autoantibodies should be considered when ICI‐related encephalitis is suspected, particularly when the characteristic MRI findings are absent. Further reports of cases are required to elucidate contributing factors.

## Author Contributions

**Takafumi Yamano:** writing – original draft. **Toshihide Yokoyama:** writing – review and editing. **Tadashi Ishida:** supervision.

## Ethics Statement

The authors declare that appropriate written informed consent was obtained for the publication of this manuscript and accompanying images. Ethical approval was not required for this case report.

## Conflicts of Interest

The authors declare no conflicts of interest.

## Data Availability

Data sharing not applicable to this article as no datasets were generated or analysed during the current study.
